# A Small Multihost Plasmid Carrying *erm*(T) Identified in *Enterococcus faecalis*

**DOI:** 10.3389/fvets.2022.850466

**Published:** 2022-05-27

**Authors:** Xing-Yun Li, Rui Yu, Chunyan Xu, Yanhong Shang, Dexi Li, Xiang-Dang Du

**Affiliations:** Department of Basic Veterinary Medicine, College of Veterinary Medicine, Henan Agricultural University, Zhengzhou, China

**Keywords:** *Enterococcus faecalis*, erythromycin, clindamycin, resistance, fitness cost, transmission

## Abstract

The aim of this study was to determine the mobile genetic elements involved in the horizontal transfer of *erm*(T) in *Enterococcus faecalis*, and its transmission ability in heterologous hosts. A total of 159 erythromycin-resistant enterococci isolates were screened for the presence of macrolide resistance genes by PCR. Whole genome sequencing for *erm*(T)-carrying *E. faecalis* E165 was performed. The transmission ability in heterologous hosts was explored by conjugation, transformation, and fitness cost. The *erm*(T) gene was detected only in an *E. faecalis* isolate E165 (1/159), which was located on a 4,244-bp small plasmid, designed pE165. Using *E. faecalis* OG1RF as the recipient strain, pE165 is transferable. Natural transformation experiments using *Streptococcus suis* P1/7 and *Streptococcus mutans* UA159 as the recipients indicated it is transmissible, which was also observed by electrotransformation using *Staphylococcus aureus* RN4220 as a recipient. The *erm*(T)-carrying pE165 can replicate in the heterologous host including *E. faecalis* OG1RF, *S. suis* P1/7, *S. mutans* UA159, and *S. aureus* RN4220 and conferred resistance to erythromycin and clindamycin to all hosts. Although there is no disadvantage of pE165 in the recipient strains in growth curve experiments, all the pE165-carrying recipients had a fitness cost compared to the corresponding original recipients in growth competition experiments. In brief, an *erm*(T)-carrying plasmid was for the first time described in *E. faecalis* and as transmissible to heterologous hosts.

## Introduction

Macrolides are a class of important natural or semisynthetic antibiotics that bind to the 50S ribosomal subunit and inhibit protein synthesis (1, 2). They have antimicrobial activity against Gram-positive and selected Gram-negative organisms (3, 4). The frequent use of macrolides in medical clinics and animal husbandry is accompanied by increased macrolide resistance, which may result in a failure of the treatment (5).

There are three ways to acquire macrolide resistance: modification of the target site, efflux pump, and drug inactivation (2, 5). Modification of the target site is mediated by 23S rRNA methylation enzymes, encoded by *erm* genes that confer resistance to macrolides; in the case of constitutive expression, they can also confer resistance to lincosamides and streptogramin B (6–11). Among the different *erm* genes currently known to occur in the different species of bacteria, *erm*(A) and *erm*(B) are most frequent (12, 13), mainly carried by a plasmid, transposons, translocatable units (TUs), and integrative and conjugative elements (ICEs) (13). Ribosomal protection gene *msr* codes for ABC-F protein confers macrolide and streptogramin B resistance. The *mef* gene codes for an efflux pump confers macrolide resistance only. Drug inactivation enzymes including phosphotransferases and esterases, which are encoded by *mph* genes and *ere* genes, respectively, confer macrolide resistance (13, 14).

Since *erm*(T) had been detected in *Lactobacillus*, it has also been described in isolates of the bacterial species *Streptococcus pyogenes, Streptococcus agalactiae, Streptococcus gallolyticus, Staphylococcus aureus, Erysipelothrix rhusiopathiae, Haemophilus parasuis*, and *Enterococcus faecium* and *Streptococcus suis* (15–22), which revealed its widespread presence. However, whether it is transmissible and whether the *erm*(T)-carrying mobile genetic elements can replicate in a heterologous host had not been fully explored. So, in this study, the presence of *erm*(T) in enterococci was investigated and the associated mobile genetic elements involved in the horizontal transfer of *erm*(T) were explored. In addition, the transmission ability and maintenance of *erm*(T)-carrying plasmid in the heterologous host was elucidated using conjugation, transformation, and fitness cost experiments.

## Materials and Methods

### Bacterial Strains and Antimicrobial Susceptibility Testing

During the routine survey for the presence of *erm*(T) in enterocci of swine origin, a total of 159 non-duplicate enterococci isolates with erythromycin MICs of no <8 mg/L were collected in July and September 2018 from anal swabs of healthy pigs at two farms in Henan Province, China.

Antimicrobial susceptibility testing (AST) was carried out by broth microdilution according to recommendations given in document M100 (Twenty-Eighth Edition) issued by CLSI (21). *S. aureus* ATCC 29213 served as the quality control strain.

*E. faecalis* OG1RF, *S. aureus* RN4220, *Streptococcus suis* P1/7, and *Streptococcus mutans* UA159 served as recipients in conjugation and transformation experiments.

### PCR Analysis

The aforementioned erythromycin-resistant enterococci strains were detected for the presence of macrolide resistance gene *erm*(T) and other resistance genes by PCR using the primers listed in Table 1 (15, 23–26). PCR products for *erm*(T) in *E. faecalis* E165 and its transconjugants and transformants were subjected to Sanger sequencing.

**Table 1:** Primer pairs used in this study.

**Gene(s)**	**Primer**	**Sequence(5^**′**^-3^**′**^)**	**Product size**	**References**
*erm*(A)	*erm*(A) forward	GCATGACATAAACCTTCA	208bp	(23)
	*erm*(A) reverse	AGGTTATAATGAAACAGA		
*erm*(B)	*erm*(B) forward	GAAAAGGTACTCAACCAAATA	639bp	(23)
	*erm*(B) reverse	AGTAACGGTACTTAAATTGTTTAC		
*erm*(C)	*erm*(C) forward	AATCGTCAATTCCTGCATAT	299bp	(24)
	*erm*(C) reverse	TAATCCTGGAATACGGGTTTG		
*erm*(F)	*erm*(F) forward	TAGATATTGGGGCAGGCAAG	178bp	(25)
	*erm*(F) reverse	GGAAATTTCGGAACTGCAAA		
*erm*(G)	*erm*(G) forward	ATAGGTGCAGGGAAAGGTCA	177bp	(25)
	*erm*(G) reverse	TGGATTGTGGCTAGGAAATGT		
*erm*(X)	*erm*(X) forward	TGACGCTGTACTCCTCATGC	410bp	(26)
	*erm*(X) reverse	GAGGAACCAGTCACCTGGAA		
*erm*(T)	*erm*(T) forward	CCGCCATTGAAATAGATCCT	478bp	(15)
	*erm*(T) reverse	GCTTGATAAAATTGGTTTTTGGA		

### Whole Genome Sequencing (WGS) and Analysis

Whole genome DNA of *E. faecalis* E165 was sequenced by the PacBio RS and Illumina MiSeq platforms (Shanghai Personal Biotechnology Co., Ltd, China). The PacBio sequence reads were assembled with HGAP4 and CANU (Version 1.6), and corrected by Illumina MiSeq with pilon (Version 1.22). The prediction of ORFs and their annotation was performed using Glimmer 3.0.

### Intraspecies Transformation and Interspecies Transformation

To investigate whether the *erm*(T)-carrying plasmid could be transferred into bacteria of the same and other species, plasmid DNA extracted from *E. faecalis* E165 was obtained by using Qiagen Mini-prep kit (Qiagen, Hilden, Germany) according to the manufacturer's protocol.

To determine the intraspecies transmissibility of the *erm*(T)-carrying plasmid pE165, conjugation experiments were performed using *E. faecalis* E165 as the donor and *E. faecalis* OG1RF as the recipient as previously described (27). Transconjugants were screened on brain heart infusion (BHI, Oxoid, British) agar supplemented with 32 mg/L rifampicin, 32 mg/L fucidin acid, and 8 mg/L erythromycin. The corresponding transconjugant was designated *E. faecalis* OG1RF-Tc.

Natural transformation experiment using the recipient *S. suis* P1/7 was performed as described to investigate the interspecies transmissibility of *erm*(T)-carrying plasmid pE165 (28). The peptide (GNWGTWVEE) was dissolved in Milli-Q water at a final concentration of 5 mM and was used as a pheromone for the transformation. The erythromycin-susceptible recipient strain *S. suis* P1/7 was grown overnight in THY broth (3 g Todd-Hewitt Broth and 2 g yeast for 100 ml, Oxoid, British) at 37°C under 5% CO_2_. The overnight culture was diluted 1:40 into pre-warmed THY broth, and grown at 37°C under 5% CO_2_ without shaking. Plasmid DNA (1.2 μg) and stock peptide (5 μl) were added when the recipient culture reached an OD_600_ between 0.035 and 0.058, and then incubated for 2h at 37°C under 5% CO_2_. The samples were diluted and plated on THA (Todd-Hewitt Broth with 1.3% agar powder, Oxoid, British) containing 8 mg/L erythromycin. The corresponding transformant was designated *S. suis* P1/7-Tm.

Natural transformation experiment with the recipient *S. mutans* UA159 was performed following procedures as previously described (29). For the screening of the transformants, THA was supplemented with 8 mg/L erythromycin. The corresponding transformant was designated *S. mutans* UA159-Tm.

The electrotransformation experiment with recipient strain *S. aureus* RN4220 was performed as described in a previous study (30). The corresponding transformant was designated *S. aureus* RN4220-Tm.

All colonies from the selective plates after incubation for 24–48 h at 37°C were further confirmed by *erm*(A), *erm*(B), and *erm*(T) gene detection (15, 23), 16S rRNA sequencing, AST, and multilocus sequence typing (MLST) following the harmonized protocols (http://www.mlst.net/). The plasmids obtained from *E. faecalis* E165, *E. faecalis* OG1RF-Tc, *S. suis* P1/7-Tm, *S. mutans* UA159-Tm, and *S. aureus* RN4220-Tm were digested by Sac I and Pac I (New England Biolabs, Inc., USA), and then southern bolt using *erm*(T) gene as the probe was performed.

### Growth Curve and Growth Competition Experiments

Growth kinetics were determined for *E. faecalis* OG1RF and *E. faecalis* OG1RF-Tc, *S. aureus* RN4220 and *S. aureus* RN4220-Tm, *S. suis* P1/7 and *S. suis* P1/7-Tm, and *S. mutans* UA159 and *S. mutans* UA159-Tm (19, 31). Volumes of 30 ml BHI broth were inoculated independently with 10^7^ CFU of OG1RF, OG1RF-Tc, RN4220, and RN4220-Tm; cultures were grown for 12 h at 200 rpm and 37°C. Volumes of 30 ml Todd-Hewitt Broth (THB, Oxoid, British) supplemented with 5% fetal calf serum were inoculated independently with 10^7^ CFU of P1/7, P1/7-Tm, UA159, and UA159-Tm; cultures were grown for 12 h at 200 rpm and 37°C. The absorbance at 600 nm was measured every hour.

The fitness cost of pE165 was determined between *E. faecalis* OG1RF and *E. faecalis* OG1RF-Tc, *S. aureus* RN4220 and *S. aureus* RN4220-Tm, *S. suis* P1/7 and *S. suis* P1/7-Tm, and *S. mutans* UA159 and *S. mutans* UA159-Tm, as previously described (31, 32), with the following modifications. OG1RF and OG1RF-Tc, RN4220, and RN4220-Tm, were cultured in BHI broth for 24h at 37°C and 200 rpm. P1/7 and P1/7-Tm, UA159 and UA159-Tm, were cultured in THB (supplemented with 5% fetal calf serum) for 24 h at 37°C and 200 rpm. Then 1 × 10^8^ CFU of recipient strain was mixed with 1 × 10^8^ CFU of corresponding transconjugant/ transformant in 30 ml antibiotic-free BHI broth/THB (supplemented with 5% fetal calf serum). The mixtures were grown at 37°C and 200 rpm and diluted at 1:100 to fresh BHI broth/THB (supplemented with 5% fetal calf serum) every 24 h. For each sample, aliquots were plated onto non-selective and erythromycin-containing BHI agar/THA (supplemented with 5% fetal calf serum) plates. The proportion of pE165-carrying strains was calculated by the number of colonies on the selective plate divided by the number of colonies on the non-selective plate.

## Results

### Identification and Characterization of *erm*(T)-Carrying Plasmid in *E. faecalis*

All erythromycin-resistant strains were investigated for the presence of the macrolide resistance genes by PCR. Of the 159 erythromycin-resistant enterococci isolates, 24(15.1%) contained solely *erm*(A), 33 (20.8%) contained solely *erm*(B), and 102 (64.2%) were positive for both *erm*(A) and *erm*(B). No strain was *erm*(C)/*erm*(F)/*erm*(G)/*erm*(X)-positive. A single *E. faecalis* strain E165 was positive for *erm*(A), *erm*(B), and *erm*(T). This is the first description of the *erm*(T) gene in *E. faecalis*.

**Figure 1:**
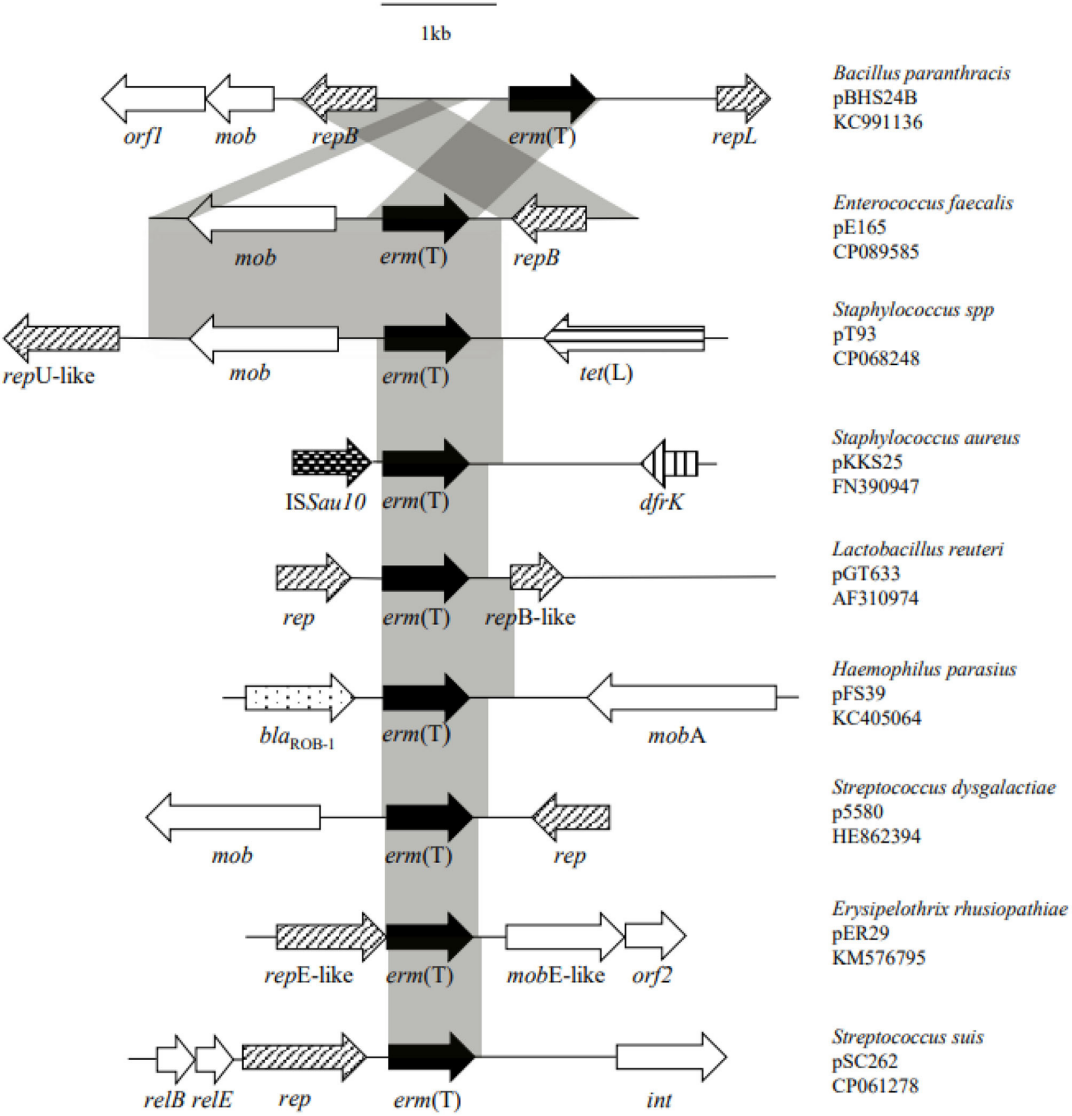
Structural comparison of the genetic environment of *erm*(T) gene located on pE165 with the genetic environment of *erm*(T) published previously.

Whole genome sequencing, assembly, and analysis for *E. faecalis* E165 showed a 4,244-bp small plasmid (designated pE165) harbored the *erm*(T) gene, with an average GC content of 33.0%. A total of three open reading frames (ORFs) encoding proteins of >100 amino acids were identified. The *erm*(T) gene coded for a 244-amino-acid (aa) protein identical to *erm*(T) of pKKS25 in *S. aureus* 25 (CAY48681.1) (18), and was highly similar (99.2% amino acid identity, 99.7% DNA sequence identity) to that of pGT633 from *Lactobacillus reuteri* 100-63 (NG_047838) (Figure 1) (32). The plasmid mobilization protein encoded by the *mob* gene from pE165 showed a high level of homology (identity≥90.6%) to that encoded by *mob* genes from *Lactococcus garvieae* (WP_207144600), *E. faecium* (HAR1670775.1), *S. suis* (WP_105139626), *S. aureus* (CCQ43999), and *Escherichia coli* (EFG1049274). The replication protein encoded by the *rep* gene from pE165 exhibited ≥99.5% identities to that encoded by *rep* genes from *E. faecalis* (HBI2052878), *Listeria monocytogenes* (HAB0665403), *S. suis* (NQK16007), *S. agalactiae* (WP_228308086), *E. faecium* (HAZ0989061), *Bacillus paranthracis* (AHN52261), *E. coli* (EFG1049261), *Lactimicrobium massiliense* (WP_108775117), and *Amylolactobacillus amylophilus* (WP_054746480), which revealed how widespread the *erm*(T)-carrying pE165-like plasmid is.

In addition, *erm*(A) gene carried by Tn*6674* was located on the chromosome. Three copies of *erm*(B) gene were located on a 65,052 bp conjugative plasmid (designated pE165-2) and the conjugative region from pE165-2 exhibits 99% DNA identity to pL15 described in an *E. faecalis* isolated from swine in Brazil (CP042214). pE165-2 also includes *tet*(M) and *tet*(L) conferring resistance to tetracyclines, *dfrG* conferring resistance to trimethoprim, *aacA-aphD* conferring resistance to aminoglycosides, and *cat* encoding chloramphenicol acetyltransferase. A 2,836 bp small plasmid that did not carry any resistance gene was also detected in *E. faecalis* E165 (designated pE165-3).

**Figure 2:**
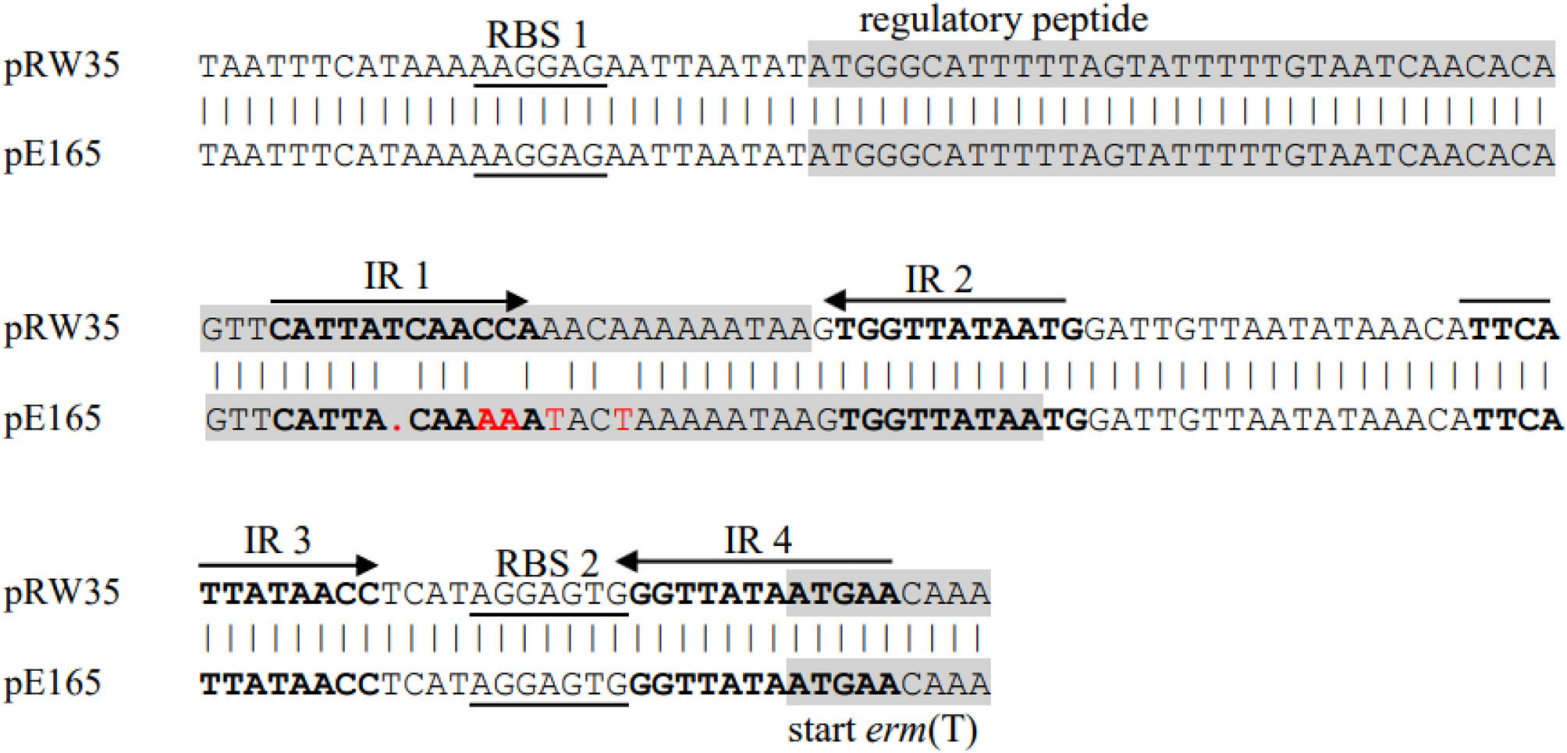
Alignment of *erm*(T) regulatory region of pRW35.seq (upper line) and *erm*(T) regulatory region of pE165.seq (lower line). Identity = 97.40%(150/154), Gap = 0.65%(1/155).

Previous studies identified a complete translational attenuator immediately upstream of the *erm*(T) gene which consisted of two pairs of inverted-repeat sequences of 12 bp each and a reading frame for a regulatory peptide of 19 aa (15, 17, 33). Inducible *erm* gene expression often required an intact translational attenuator, while deletions or duplications that appeared in the regulatory region would cause constitutive *erm* gene expression (34). A comparison of the *erm*(T) regulatory region of pE165 with that of plasmids pRW35 (EU192194) revealed that the *erm*(T) regulatory region of pE165 had four bp point mutations and one bp deletion in the regulatory peptide ORF. This single nucleotide deletion resulted in a frame shift mutation, which extended the reading frame for the regulatory peptide from 19 aa to 22 aa (Figure 2). In addition, *erm*(T) regulatory region of pE165 was compared to those of pSC262 and pUR2940 with mutations in previous reports (22, 35), and the results are shown in Supplementary Figures S1, S2.

### The *erm*(T)-Carrying Plasmid Is Transmissible

Intraspecies transmissibility of pE165 was investigated by conjugation experiments using *E. faecalis* E165 as the donor and *E. faecalis* OG1RF as the recipient. The transconjugant OG1RF-Tc was successfully obtained on the selective plates with the transfer frequencies of 1.5 × 10^−5^. PCR assay was used for the detection of *erm*(T), *erm*(A), and *erm*(B) in transconjugant, and the results revealed that only *erm*(T) gene was transferred into the recipient. Minimum inhibition concentrations (MICs) of *E. faecalis* E165, OG1RF, and OG1RF-Tc were determined and are shown in Table 2. Compared to *E. faecalis* OG1RF, OG1RF-Tc displayed a higher erythromycin MIC (>512 mg/L) and higher clindamycin MIC (256 mg/L).

**Table 2:** MICs of *erm*(T)-carrying *E. faecalis* E165, recipient strains *E. faecalis* OG1RF, *S. aureus* RN4220, *S. suis* P1/7, *S. mutans* UA159, and corresponding transconjugants/transformants.

**Strains**	**MICs (mg/L)** ^**a**^							
	**ERY**	**LIN**	**CLI**	**CHL**	**FFC**	**TET**	**LND**	**CIP**
E165	>512	>512	512	128	64	64	4	32
OG1RF	1	32	16	4	2	<1	0.5	2
OG1RF-Tc	>512	512	256	4	2	<1	0.5	2
RN4220	<1	<1	<1	2	2	<1	0.25	<1
RN4220-Tm	>512	512	128	2	2	<1	0.25	<1
P1/7	<1	<1	<1	1	1	<1	0.25	1
P1/7-Tm	512	64	32	1	1	<1	0.25	1
UA159	<1	<1	<1	2	1	<1	0.25	1
UA159-Tm	>512	512	256	2	1	<1	0.25	1

a *ERY, erythromycin; LIN, lincomycin; CLI, clindamycin; CHL, chloramphenicol; FFC, florfenicol; TET, tetracycline; LND, linezolid; CIP, ciprofloxacin*.

Interspecies transmissibility of pE165 was investigated by natural transformation using *S. suis* P1/7 and *S. mutans* UA159 as the recipients and electrotransformation using *S. aureus* RN4220 as the recipient. Transformants P1/7-Tm, UA159-Tm, and RN4220-Tm were successfully obtained, and the transfer frequencies were 0.63 × 10^2^ μg^−1^,2.1 × 10^2^ μg^−1^ and 4.9 × 10^4^ μg^−1^. The transformants were confirmed by AST and sequencing of 16S rRNA. PCR assay also revealed that only *erm*(T) could be detected in these transformants. MICs of *E. faecalis* E165, *S. suis* P1/7, *S. mutans* UA159, *S. aureus* RN4220, and their transformants are shown in Table 2. *S. suis* transformant P1/7-Tm displayed a higher erythromycin MIC (>512 mg/L) and a higher clindamycin MIC (32 mg/L) compared with *S. suis* P1/7. *S. mutans* transformant UA159-Tm displayed higher erythromycin MIC (>512 mg/L) and clindamycin MIC (256 mg/L) compared with *S. mutans* UA159. *S. aureus* transformant RN4220-Tm displayed higher erythromycin MIC (>512 mg/L) and higher clindamycin MIC (128 mg/L) compared with *S. aureus* RN4220.

**Figure 3:**
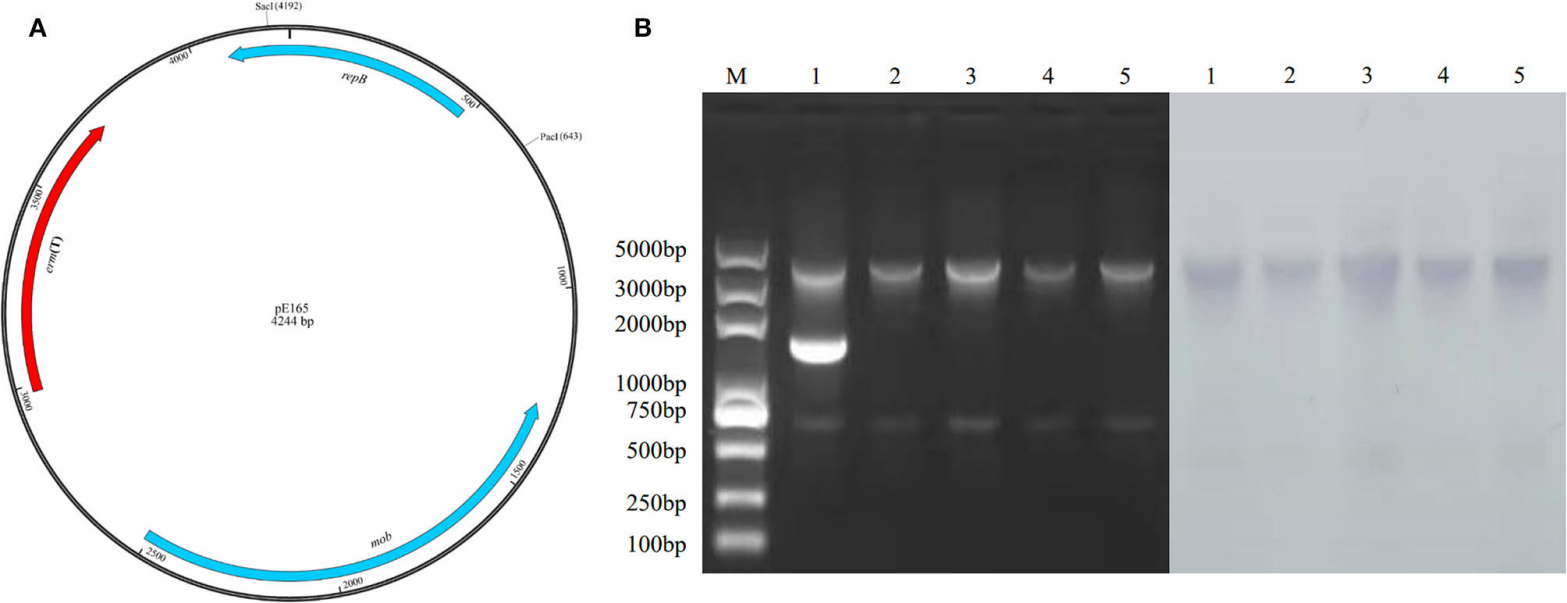
**(A)** Restriction enzyme cutting sites of Sac I and Pac I on pE165. **(B)** Restriction enzyme-digested plasmid profiles treated by Sac I, Pac I, and southern blot using *erm*(T) gene as the probe. M: marker 1; *E. faecalis* E165, 2; *E. faecalis* OG1RF-Tc, 3; *S. mutans* UA159-Tm, 4; *S. suis* P1/7-Tm, 5; *S. aureus* RN4220-Tm. The upper and lower bands were the fragments of pE165 digested by Sac I and Pac I. The middle band in line 1 was supercoiled pE165-3.

The physical map of pE165 and restriction enzyme-digested plasmid profiles of the plasmids from *E. faecalis* E165, transconjugant *E. faecalis* OG1RF-Tc, transformants *S. suis* P1/7-Tm, *S. mutans* UA159-Tm, and *S. aureus* RN4220-Tm are shown in Figure 3. The results indicated that only pE165 can transfer into the recipient strains and pE165 can replicate in heterogenous hosts. The result of the southern bolt revealed that *erm*(T) gene was located on pE165 in heterogenous hosts. The results of AST indicated that pE165 can constitutively express erythromycin- and clindamycin- resistance phenotype in heterogenous hosts.

### Fitness Cost

**Figure 4:**
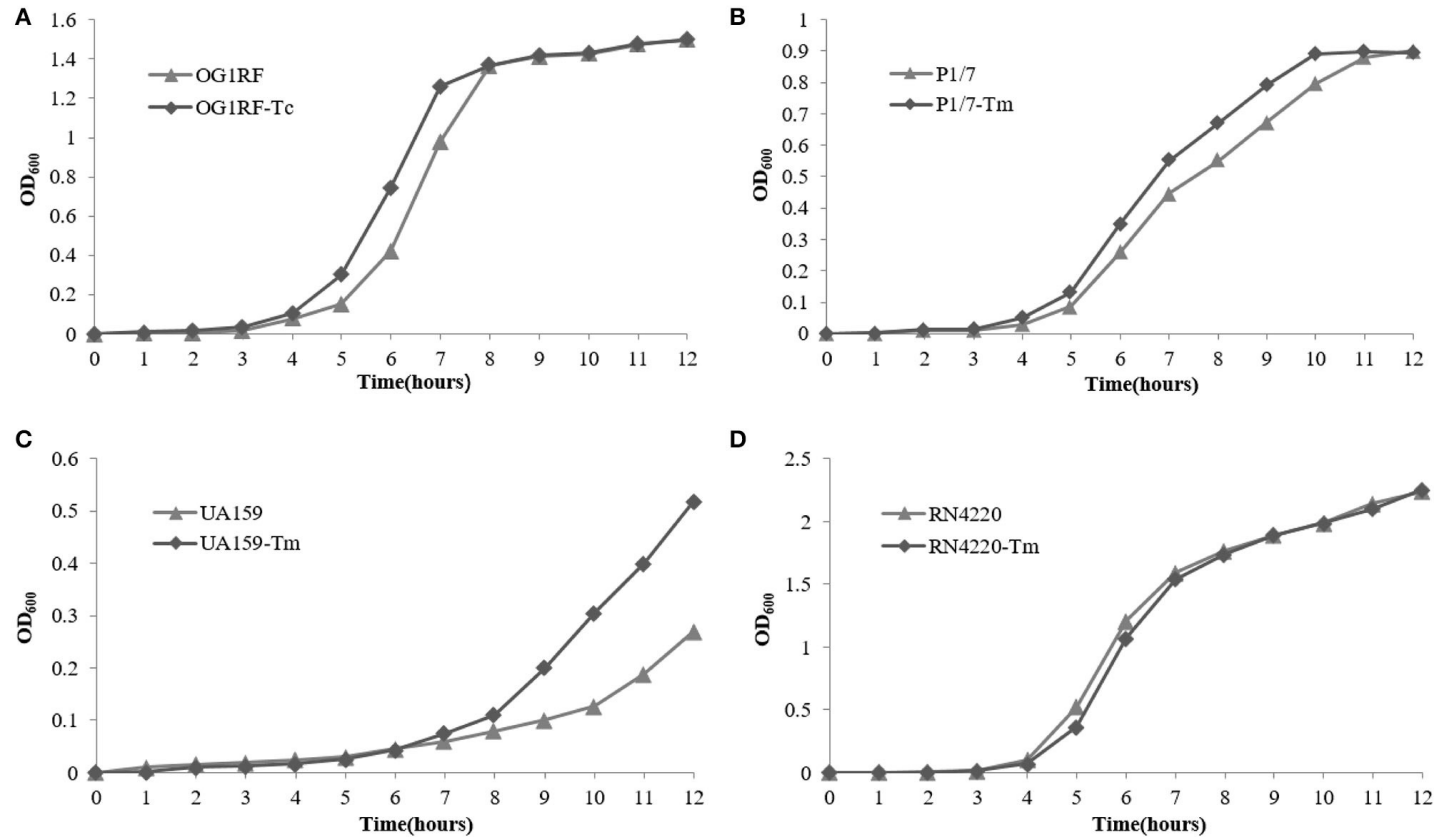
Fitness cost of pE165. **(A–D)** Comparison of growth kinetics of *E. faecalis* OG1RF and *E. faecalis* OG1RF-Tc, *S. aureus* RN4220 and *S. aureus* RN4220-Tm, *S. suis* P1/7 and *S. suis* P1/7-Tm, *S. mutans* UA159 and *S. mutans* UA159-Tm in the absence of erythromycin.

The growth curve of *E. faecalis* E165, *S. suis* P1/7, *S. mutans* UA159, *S. aureus* RN4220, and their transconjugants and transformants in the absence of erythromycin are shown in Figure 4. The results showed that no significant fitness burden for pE165-carrying transconjugants and transformants was observed compared with the recipient strains in the absence of selective pressure.

**Figure 5:**
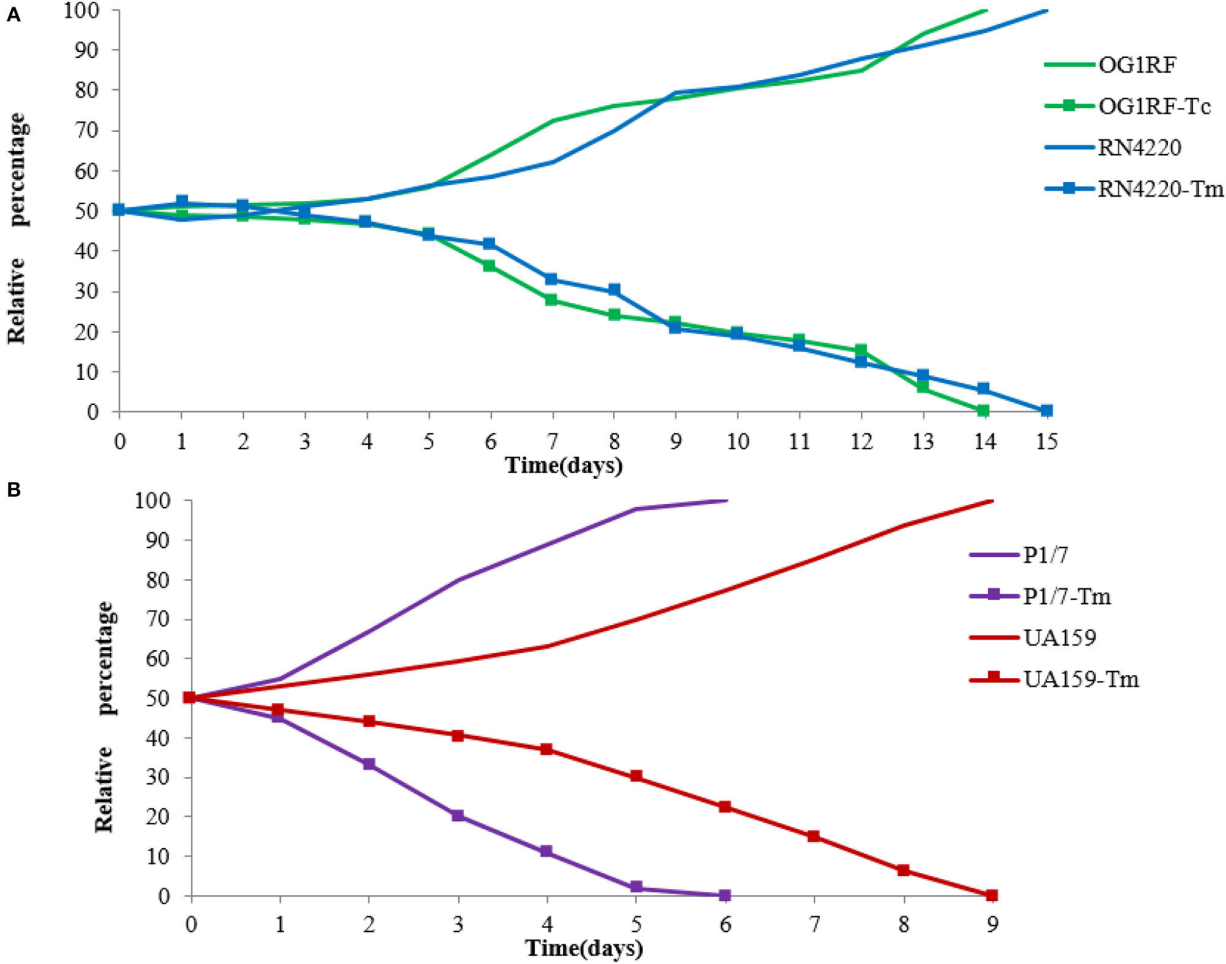
**(A)** Growth competition experiments between *E. faecalis* OG1RF and *E. faecalis* OG1RF-Tc, *S. aureus* RN4220 and *S. aureus* RN4220-Tm. **(B)** Growth competition experiments between *S. suis* P1/7 and *S. suis* P1/7-Tm, *S. mutans* UA159 and *S. mutans* UA159-Tm. The initial ratio of the recipient strain carrying pE165 to the original recipient strain was 1:1. The results above were averagely calculated from four independent experiments.

Competition experiments can offer a more discriminative and precise measurement of fitness, and the competitive disadvantage of the fitness burden caused by pE165 can be reflected during all the phases of the growth cycle and in successive cycles. During the competition experiment between *E. faecalis* OG1RF and OG1RF-Tc, from day 1 on, a successive decrease in the proportion of *E. faecalis* OG1RF-Tc was observed, and all the colonies tested were pE165 free on day 14 (Figure 5A). From day 1 on, a fast and constant decrease in the proportion of *S. suis* P1/7-Tm was observed and all the strains were tested pE165 free on day 6 (Figure 5B). In the process of competition experiment between *S. mutans* UA159 and UA159-Tm, an obvious decrease in the proportion of UA159-Tm was observed. On day 9, all the colonies tested were pE165 free (Figure 5B). For the result of the competition experiment between *S. aureus* RN4220 and RN4220-Tm, it had an obvious decrease from day 3 on, and RN4220-Tm could not be detected on day 15 (Figure 5A). The above results suggested that all the pE165-carrying transconjugants and transformants had a fitness cost compared to the recipient strains without pE165, but the fitness cost among the different transconjugants and transformants differed.

## Discussion

Since *erm*(T) has been reported from *Lactobacillus reuteri* (33), it had been described in various genera: *Lactobacillus, Streptococcus* (*S. pyogenes, S. suis*), *S. aureus, E. faecium*, and other gram-positive bacteria including *E. rhusiopathiae* (KM576795.1), even in gram-negative bacteria such as *H. parasuis* (KC405064.1) and *Klebsiella pneumonia* (CP040837.1), revealing its widespread presence. Mobile genetic elements play a crucial role in the horizontal gene transfer of *erm*(T), including plasmids (15, 16, 18, 20, 35–37),transposons (38, 39), and insert sequence (17). *erm*(T)-positive plasmids of *S. agalactiae* could efficiently be transferred into group B Streptococcus and in the *E. faecalis* recipient strain (16).

In this study, *erm*(T)-positive plasmid pE165 was identified in a *E. faecalis* strain E165. Whole-genomic sequencing for E165 was performed. The genetic environment of *erm*(T) in this study was then analyzed by comparing it with similar *erm*(T) genetic environments published previously (19, 20, 33, 35, 39, 40). The homology analysis of the *rep* and *mob* genes located on pE165 suggested that this plasmid had the potential ability to transfer into enterococci and other species. Transformation experiments confirmed that pE165 was successfully transferred into *Enterococcus, Streptococcus*, and *Staphylococcus*. Elevated MICs of erythromycin and clindamycin were conferred by *erm*(T) in the recipient strains. According to recommendations given in CLSI, inducible expression of *erm*(T) cannot produce clindamycin resistance unless it is induced by erythromycin (41). In this study, the inducible clindamycin resistance tests indicated that the transconjugant *E. faecalis* OG1RF-Tc, transformants P1/7-Tm, UA159-Tm, and RN4220-Tm were resistant to both erythromycin and clindamycin (Table 2), which revealed the expression of *erm*(T) in these strains was constitutive. This is also in agreement with the observation that deletions or duplications appeared in the regulatory region of *erm*(T) in these strains.

It can be found that the proportion of pE165-carrying strains constantly decreased until undetectable in the competition experiments between transformants and original recipients performed by successive culturing in the absence of antibiotic pressure. Although plasmids can mediate the horizontal transmission of resistance genes between bacteria and facilitate their adaptation to the pressure of antibiotics, they also entail a metabolic burden that reduces the competitiveness of the plasmid-carrying clone in the absence of selection (42). Acquisition and maintenance of a plasmid are directly associated with fitness effects on the recipient strain. The constitutive expression of *erm*(T) will produce a burden (fitness cost) in the recipient strain, and the low prevalence of *erm*(T) gene in many genera of bacteria may be explained in this way.

## Conclusions

The *erm*(T) gene was first reported in an *E. faecalis* strain. A 4244 bp *erm*(T)-positive plasmid pE165 was characterized. The transmissibility of pE165 was investigated between intra- and inter-species. The presence of pE165 greatly elevated the MICs of erythromycin and clindamycin which indicated the expression of *erm*(T) in the recipient strains was constitutive. Although the fitness cost showed us this plasmid reduced the competitiveness of the host strain, the potential possibility of dissemination of *erm*(T) among species of bacteria should not be ignored.

## Data Availability Statement

The datasets presented in this study can be found in online repositories. The names of the repository/repositories and accession number(s) can be found below https://www.ncbi.nlm.nih.gov/, CP089585.

## Author Contributions

X-DD and DL designed the research and supervised the study. X-YL, RY, CX, and YS performed the experiments and analyzed the data. X-YL, RY, and X-DD wrote the manuscript. All authors revised the manuscript and approved the final version for submission.

## Funding

This work was supported by grants from the Program for Innovative Research Team (in Science and Technology) at the University of Henan Province (No. 18IRTSTHN020).

## Conflict of Interest

The authors declare that the research was conducted in the absence of any commercial or financial relationships that could be construed as a potential conflict of interest.

## Publisher's Note

All claims expressed in this article are solely those of the authors and do not necessarily represent those of their affiliated organizations, or those of the publisher, the editors and the reviewers. Any product that may be evaluated in this article, or claim that may be made by its manufacturer, is not guaranteed or endorsed by the publisher.
