# Supplementary material for: A Small Multihost Plasmid Carrying erm(T) Identified in Enterococcus faecalis
Source: Front Vet Sci. 2022 May 27;9:850466. doi: 10.3389/fvets.2022.850466 (PMC9197182; doi:10.3389/fvets.2022.850466)
Supplement: Supplementary file 1 [file Data_Sheet_1.pdf]

Alignment of *erm*(T) regulatory region of pE165.seq (upper line) and *erm*(T) regulatory region of pUR2940.seq (lower line). Identity= 73.68%(112/152), Gap= 10.06%(17/169)

```
------------------

pE165     TAATTTCATAAAAAGGAGAA...............TTAATATATGGGCATTTTTAGTATT
           |||| ||  | ||    ||               ||| ||| |        ||    |
pUR2940   CAATTGCAGTATAAATTTAACAGTCGTTCATCATGTTATTATTTATCAGAGCTTGTGCTA

pE165     TTTGTAATCAACACAGTTCATTACAAAAATACTAAAAATAAGTGGTTATAATGGATTGTT
          |   || || |      | ||  |||  | || |||||||||||||||||||||||||||
pUR2940   TAATTATTCTAATTTCATAAT..CAACCAAACAAAAAATAAGTGGTTATAATGGATTGTT

pE165     AATATAAACATTCATTATAACCTCATAGGAGTGGGTTATAATGAACAAA
          |||||||||||||||||||||||||||||||||||||||||||||||||
pUR2940   AATATAAACATTCATTATAACCTCATAGGAGTGGGTTATAATGAACAAA
                                                 start erm(T)
```

FIGURE S1 | Alignment between the regulatory region of *erm*(T) on pE165 and that on pUR2940.

Alignment of *erm*(T) regulatory region of pE165.seq (upper line) and *erm*(T) regulatory region of pSC262.seq (lower line). Identity= 52.29%(80/153), Gap= 1.92%(3/156)

____

```
pE165   TAATTTCATAAAAAGGAGAATTAATATATGGGCATTTTTAGTATTTTTGTAATCAACACA
            |    ||   |  ||  ||||||    | |  ||||      ||  ||       |
pSC262  ATTGTCATGAAGCTGATGAGCTAATATGATAGAACGTTTATCTGAATTTGAAGATGTG.A

pE165   GTTCATTACAAAAATACTAAAAATAAGTGGTTATAATGGATTGTTAATAT..AAACATTC
        |      |  ||| |||||     | |   ||| |||||  |       |  ||||  |
pSC262  GGGATAGAAGAAACTACTATGCCAAGGGAATTAAAATGGCATTGCGGATTGAAAACTATT

pE165   ATTATAACCTCATAGGAGTGGGTTATAATGAACAAA
         | ||||    |||||||||||||||||||||||||
pSC262  CTGATAATGAGATAGGAGTGGGTTATAATGAACAAA
                                   start erm(T)
```

FIGURE S2 | Alignment between the regulatory region of *erm*(T) on pE165 and that on pSC262.
